# Tablet-Based Puzzle Game Intervention for Cognitive Function and Well-Being in Healthy Adults: Pilot Feasibility Randomized Controlled Trial

**DOI:** 10.2196/46177

**Published:** 2023-11-01

**Authors:** Prabitha Urwyler, Rajnish Kumar Gupta, Michael Falkner, Joel Niklaus, René Martin Müri, Tobias Nef

**Affiliations:** 1 Gerontechnology & Rehabilitation Group, University of Bern, Bern, Switzerland; 2 ARTORG Center for Biomedical Engineering Research, University of Bern, Bern, Switzerland; 3 Department of Neurology, University Neurorehabilitation, University Hospital Bern, Inselspital, University of Bern, Bern, Switzerland; 4 Department of Psychology, Manipal University Jaipur, Jaipur, India; 5 University Hospital of Old Age Psychiatry and Psychotherapy, University of Bern, Bern, Switzerland

**Keywords:** puzzle games, aging, cognitive assessment, visual attention, adults, elderly, well-being, randomized controlled trial, RCT, older adult

## Abstract

**Background:**

Promoting cognitive health is key to maintaining cognitive and everyday functions and preventing the risk of cognitive impairment or dementia. Existing scientific evidence shows the benefits of various training modalities on cognition. One way to promote cognitive health is through engagement in cognitive activities (eg, board and video games).

**Objective:**

This study aims to investigate the benefits of dynamic adaptive casual puzzle games on cognitive function and well-being in healthy adults and older people.

**Methods:**

A total of 12 adults and older people (female participants: n=6; mean age 58.92, SD 10.28 years; range 46-75 years) were included in this pilot randomized controlled trial. This study used a crossover design with two phases (8 weeks each) and three measurement waves (pretest, midtest, and posttest). The participants were randomly allocated either to the control or experimental group. In the control group, participants read newspapers between the pre- and midtest, then switched to cognitive training with puzzle games. In the experimental group, the interventions were reversed. Baseline measurements (pretest) were collected before the intervention. The interventions were delivered on tablet computers and took place unsupervised at participants’ homes.

**Results:**

The outcome measures included global cognitive function, higher cognitive function, and emotional well-being at 3 time points (pretest, midtest, and posttest) using standardized neuropsychological tests. The participants showed improvements in their visual attention and visuospatial measures after the puzzle game intervention.

**Conclusions:**

The study showed that digital games are a feasible way to train cognition in healthy adults and older people. The algorithm-based dynamic adaption allows accommodations for persons with different cognitive levels of skill. The results of the study will guide future prevention efforts and trials in high-risk populations.

## Introduction

The world’s older adult population is increasing in both size and proportion. By 2050, the world’s population of older people will nearly double [1]. The aging population inevitably requires better health care services for age-related diseases and the prevention of physical and cognitive health decline. Age-related cognitive decline affects memory, attention, orientation, perception, and executive and motor function that impact everyday activities and quality of life [2,3]. Considering population aging as a dominant worldwide phenomenon, preventing or reversing cognitive decline are clinical and public health priorities [3].

The use of video games for training in strategic control in structured conditions may enhance the cognition of both adults and older people [4-7]. Ample research suggests that playing video games enhances a variety of cognitive skills such as visual selective attention, speed of processing, memory, reasoning, problem-solving, learning, and executive functioning [8-13]. The use of video games for cognitive assessment may facilitate reliable, personalized, and cost-effective tests [14]. Video games provide a variety of performance data (eg, number of errors, reaction time, and other task-based measures) that can make cognitive function assessments more accurate [15].

Traditional cognitive training programs require an in-person meeting, which entails setting a meeting location, traveling, and scheduling an appointment. For traditional training programs, recruiting older adults who are homebound or live in assisted living is more challenging. Computer-based cognitive interventions are potentially a more efficient alternative to traditional training programs that enhance their users’ accessibility, affordability, and applicability. Computerized training provides real-time feedback and can be personalized, restoring users’ activity engagement and motivation for the program [16,17] and eliminating staff load and training requirements, as well as errors and deliverer and observational biases in patient performance and performance anxiety. For a long-term effect (improvement in a broad range of cognitive domains), it is important to maximize player engagement.

Computerized training can be deployed either as virtual reality games, augmented reality games, serious games, or casual video games (CVGs). CVGs are primarily designed for entertainment [18] and share features that closely match recommendations for video games for older adults [19]. CVGs have simple and minimized game elements, rules, and goals (simplicity); can easily be stopped and replayed, are error-forgiving, and use flexible difficulty levels (flexibility); and allow players to quickly learn the game (accessibility). One of the main advantages of using CVG-based tools is in their motivational properties, which are crucial in making the interaction enjoyable and engaging [20]. Previous studies have demonstrated artificial intelligence (AI)–based interventions as a promising tool for enhancing cognition, quality of life, or well-being among older adults [21-23]. A recent large-scale study showed that regular engagement in Sudoku and similar puzzles represent a cognitively enriching leisure activity that prevents and delays age-related cognitive decline [24]. Several studies have suggested puzzle video games are sensitive to the cognitive or motor alternations of normal aging [25,26]. In particular, the mazelike numberlink and match-3 puzzle video games’ performance was shown as a strong predictor of assessing cognitive or motor variabilities in older adults [25,27]. These games can be varied in difficulty to match the user’s level of cognitive ability and can help prevent practice effects during repeated administration and reduce ceiling and flooring effects by continuously matching the task difficulty to the participant’s cognitive ability [28,29].

The study aimed to conduct a pilot randomized controlled trial (RCT) to evaluate the potential benefits of the puzzle game intervention in healthy adults including older adults. The primary objective was to examine whether a puzzle game supported by AI personalization significantly improves attentional function (visual search attention) and leads to in-game learning effects. Other secondary objectives were to investigate improvements in further cognitive outcome measures proposed to be engaged by the puzzle game (attention, processing speed, working memory, and spatial reasoning) and the efficacy of the puzzle game intervention in reducing symptoms of depression, anxiety, and stress and improving everyday function and quality of life. First, we expected significant improvement in attentional and executive function (near transfer) and other cognitive functions engaged by the game (far transfer). Second, we generalized that well-being (mood and stress) will show significant improvement.

## Methods

### Ethical Considerations

Ethics approval was granted by the University of Bern Ethics Committee and the Ethics Committee Northwest/Central Switzerland (ID 2016-01281). The study was carried out following the current version of the Declaration of Helsinki and the Good Clinical Practice guidelines. A written informed consent form was sought from each participant.

### Participants

The sample included healthy adults and older adults (N=12; female: n=6, 50%; mean age 58.92, SD 10.28 years; range 46-75 years) recruited from the local community. The inclusion criteria for participation were adults aged between 45 and 75 years and with a Montreal Cognitive Assessment (MoCA) score ≥24. The exclusion criteria for participation were any previous history of comorbid neurological or psychiatric deficits; any previous diagnosis of mild or major neurocognitive disorder; and insufficient coordinative, motor, and perceptual ability to handle a tablet computer. All participants had normal or corrected-to-normal vision.

The data of a participant (ID 09) was excluded due to the incomplete measurement of the participant’s cognitive function and emotional well-being at the 3 time points (pretest, midtest, and posttest).

### Study Design

**Figure 1.**
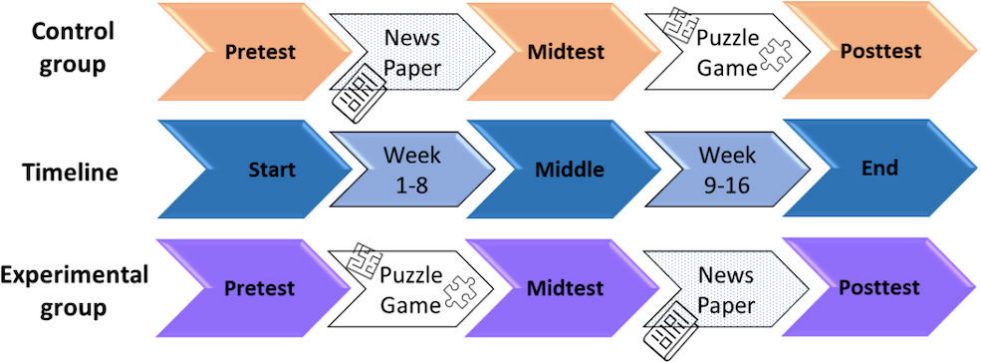
Study timeline. Participants were randomly allotted either to the control or experimental group. To test the baseline, a pretest was conducted for each participant; after 8 weeks, a midtest was conducted; and then after 8 weeks of the second intervention, a posttest was performed. In the experimental group, interventions were reversed.

The pilot was designed as a 16-week randomized controlled trial using a crossover design with two phases (8 weeks each) and three waves (pretest, midtest, and posttest) of measurement. The participants were randomly allocated either to the control or experimental group. Baseline measurements (pretest) were collected before the intervention. In the control group, participants read newspapers at least 3 times a week for 8 weeks between the pre- and midtest, then switched to cognitive training with puzzle games after the midtest. In the experimental group, the interventions were reversed compared to the control group. The interventions were delivered on tablet computers (10.2” Apple iPad 2019 model, 32 GB, 4G edition [for SIM card], Apple Inc., Cupertino, CA) and took place unsupervised at participants’ homes (Figure 1). After completing recruitment formalities, participants were given an introduction to the study followed by a training session of both games consisting of 4 trials each of three grid sizes (4 × 4, 5 × 5, and 6 × 6). In addition, a manual/handbook was given to each of the participants.

At 3 time points (pretest, midtest, and posttest), global cognitive function, higher cognitive function, and emotional well-being were assessed using standardized neuropsychological tests (computerized visual-scanning Test of Attentional Performance [TAP] task [30], MoCA [31], Trail Making Test [TMT] [32], and Snellgrove Maze Test [33]) and questionnaires (Profile of Mood States [34-36], State-Trait Anxiety Inventory [37,38], and a quality of life questionnaire [39]). To remove bias in too-close measurements, alternative versions of MoCA were used for the pretest, midtest, and posttest. Additionally, information on the cognitive load (NASA Task Load Index [40]), motivation (adapted version of Intrinsic Motivation Inventory [IMI] [41]), and familiarity with using a tablet (adapted Tablet Familiarity Questionnaire [42]) was collected at the end of the study. All assessments were administered in paper-and-pencil format except for the computerized visual-scanning TAP task presented on a laptop. In the visual-scanning TAP task, participants actively scanned a 5 × 5 matrix and indicated whether a specific target stimulus (square with a top opening) was present among 3 types of similar distractor stimuli (squares with openings on the left, right, or bottom).

### Newspaper-Reading Task

The participants were instructed to read a newspaper on the iPad for about 8 weeks at least 3 times a week for about 20 minutes a day. Some of the locally known news apps (BBC, Tages-Anzeiger, 20 Minuten, NZZ, etc) were preinstalled, and participants were free to choose a news app of their choice.

### Puzzle Game Task

Participants were instructed to play both puzzle games (numberlink and match-3), delivered to them on an iPad, a minimum of 3 times per week (maximum of 10 min/game). A time tracker in the game limited the participants’ daily training time to 20 minutes, while a scoreboarder displayed stars corresponding to the number of levels completed by the participants. The training duration of 20 minutes a day was selected to avoid any fatigue or complaints of addiction. We also hypothesized that playing for shorter sessions over a longer study duration had better transfer effects and avoided early ceiling effects. Previous studies on game interventions in adults also used a similar training duration [43,44]. To begin with, participants were assigned an initial difficulty level depending on their scores from the pretest. The difficulty level for the next day was updated depending on the game performance of the participants on the previous day by the AI-based method.

**Figure 2.**
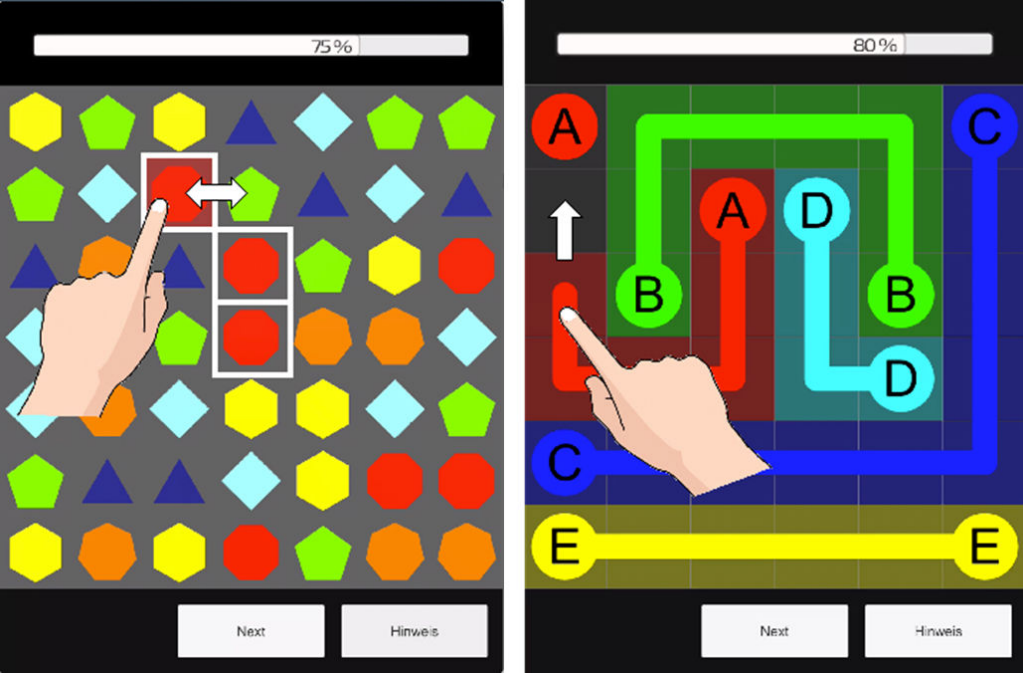
Left: A match-3 puzzle is played by swapping objects to make a row of three identical objects. Right: A numberlink puzzle is played by finding paths to connect pairs of objects [45].

The match-3 puzzle [27] (Figure 2 left) requires visual search, visuospatial working memory, and pattern recognition skills to swap game objects into a row of three identical objects. The numberlink puzzle [25] (Figure 2 right) requires visuospatial, visuo-constructional, and executive (planning, foresight, and reasoning) functions to find paths between identically colored pairs of game objects, filling all empty cells. A range of difficulty levels for both games was implemented using the height and width of the puzzle board (from 4 × 4 to 8 × 8) and the number of unique game objects (from 4 to 8).

### Puzzle Game Difficulty

To dynamically adjust the puzzle game difficulty during the cognitive intervention, an AI-based method was developed. We hypothesized that the AI method could continuously model the participant’s ability through a multidimensional combination of puzzle difficulty parameters (ability assessment) and could adjust the puzzle difficulty as the participant progresses within the intervention (adaptivity assessment). To begin with, the participant’s initial level is set using predefined variables collected at the pretest before the game intervention. When a puzzle level is solved, the AI service receives the solving time (state, *t_m_*) and decides whether this lies within a threshold (reward). The AI agent then learns whether to increase or decrease the puzzle’s difficulty.

The model is built on a database of input features, a regressor, the time predictor, and the level predictor. The end points are *match_three/ putInitialLevel*, *match_three/ putNextLevel*, *number_link/ putInitialLevel*, and *number_link/ putNextLevel*. The database of input features is a collection of sorted participant training data and difficulty level (determined by the set size and the number of gems/tiles) data for the two games. The participants’ data were cleaned and prepared with pandas 1.0.3 and numpy 1.18.1. The regressor was selected depending on the adjusted *R*^2^ value on the training data set. Accordingly, a voting regressor was chosen for the match-3 puzzle and an MLPRegressor for the numberlink puzzle. The features of just completed or played levels are sent to a timing predictor to calculate the predicted time *t_p_*. The level predictor uses solved time *t_m_* and predicted time *t_p_* as inputs to provide an output of –1 (retreat one level), 0 (stay on the same level), or 1 (advance one level).


$$f\left(t_{m},t_{p}\right)=\left\{ \begin{array}{l} -1,\;\;if\;t_{m}>t_{p}+\;c\;\times \;\sigma ,\\ \;\;1,\;\;if\;t_{m}<\;t_{p}\;-\;c\;\times \;\sigma ,\\ \;\;0,\;\;otherwise. \end{array}\right.$$

where *c* is a constant between 0 and 1, and σ is the SD of all the games played on the given difficulty level in the data set.

### Implementation

The AI service was implemented in Python 3.8.2 (Python Software Foundation) and deployed on a CentOS cloud instance. Every entity in the architecture of the AI service corresponds to a Python file. The AI service is queried via a representational state transfer (REST) application programming interface (FastAPI 0.52.0 and uvicorn 0.11.3). The pilot RCT tablet computers were equipped with SIM cards to ensure a continuous connection with the AI server. A mail-alerting service using the free tier of Mailgun was implemented to follow up with the daily progress of the participants’ gameplay.

### Data Analysis

Time-based performance indicators such as overall solving time (minutes), average target search time (seconds), and processing time per item (seconds) were calculated from the files stored on the tablet computer. The improvement in the match-3 puzzle was calculated as the slope of the search time over the intervention period; similarly, the slope of path completion time was used for the numberlink puzzle.

Statistical analysis was performed in SPSS v20.0 (IBM Corp). The normality of all values of interest was checked using the Shapiro-Wilk test [46]. An α value of .05 was used to determine significance. To evaluate the intervention effect on participants’ cognitive function and emotional well-being at 3 time points (pretest, midtest, and posttest), repeated-measure ANOVA was performed. Paired *t* tests were used to compare the visual search attention task (visual-scanning TAP) before and after the intervention wave. Correlation analyses were used to examine the intervention performance and efficiency and the cognitive and emotional measures.

## Results

The demographics and characteristics of the participants included in this pilot study are shown in Table 1.

**Table 1.** Participant demographics and characteristics.

Group and subject ID		Age (years)	Gender	Handedness	MoCA^a^	TMT^b^-A (s)	TMT-B (s)	SMT^c^ (s)
**Control group**								
	01	75	Male	Right	26	38	119	27
	02	51	Female	Left	28	29	67	13
	03	46	Female	Left	29	23	46	10
	04	66	Female	Right	29	43	95	31
	05	53	Female	Right	30	18	43	13
	06	56	Male	Left	28	45	71	11
	07	74	Male	Right	25	51	268	34
**Experimental group**								
	08	48	Female	Right	29	29	49	13
	10	59	Male	Right	24	22	122	18
	11	72	Male	Right	25	41	120	29
	12	52	Female	Right	28	20	45	15
	13	55	Male	Right	30	28	57	16

a MoCA: Montreal Cognitive Assessment (score: 1-30).

b TMT: Trail Making Test. TMT-A is the selective attention test set. TMT-B is the divided attention test set.

c SMT: Snellgrove Maze Task.

**Figure 3.**
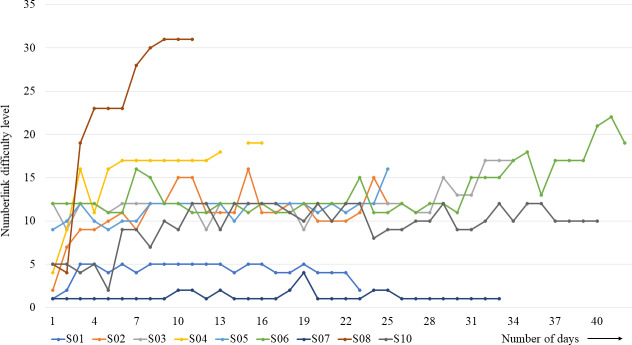
Numberlink difficulty level of subjects S01 to S10 over intervention duration.

The participants assigned to the control group read newspapers (20 min/d) between the first and second waves of assessments, then switched to cognitive training with the match-3 (10 min/d) and numberlink (10 min/d) puzzles after the second wave of assessments. In the experimental group, the interventions were reversed compared to the control group. The AI server logged every request sent from the iPad to the server. Figure 3 shows the duration of the game training intervention for the numberlink puzzle and the progress in the difficulty level.

There was a significant difference between the pre- and postintervention primary outcome measure (visual-scanning TAP; total trials: mean 5.04, SD 0.82 vs mean 4.41, SD 0.63; *t*_11_=3.5; *P*=.005; trials with targets present: mean 4.98, SD 0.76 vs mean 4.41, SD 0.73; *t*_11_=4.23; *P*=.001). No significant differences were found with the measures of global cognition (MoCA), selective (TMT-A completion time) and divided (TMT-B completion time) attention, mood disturbances, anxiety, and quality of life when compared between the 3 time points (pretest, midtest, and posttest), as shown in Table 2. However, there was a significant difference in the MoCA visuospatial executive subscore for the midtest and posttest (mean 3.83, SD 1.03 vs mean 4.58, SD 0.67; *P*=.04).

**Table 2.** Neuropsychological, emotional, and quality of life scores.

Variables and time point		Score, mean (SD)	*P* value^a^
**Montreal Cognitive Assessment (score: 1-30)**			.40
	Pre	27.58 (2.06)	
	Mid	26.92 (2.43)	
	Post	27.58 (2.64)	
**Trail Making Test–A^b^ (s)**			.44
	Pre	32.25 (10.96)	
	Mid	31.50 (12.99)	
	Post	30.08 (10.88)	
**Trail Making Test–B^c^ (s)**			.13
	Pre	91.83 (63.43)	
	Mid	63.75 (30.36)	
	Post	66.75 (27.48)	
**Snellgrove Maze Task (s)**			.02
	Pre	19.17 (8.59)	
	Mid	19.75 (9.55)	
	Post	16.50 (8.12)	
**Total mood disturbance (from Profile of Mood States)**			.35
	Pre	47.58 (7.34)	
	Mid	47.25 (6.05)	
	Post	43.42 (10.83)	
**Trait anxiety**			.51
	Pre	30.48 (17.11)	
	Mid	27.46 (14.94)	
	Post	24.53 (11.76)	
**State anxiety**			.99
	Pre	26.11 (14.38)	
	Mid	25.68 (18.04)	
	Post	25.72 (22.77)	
**EQ-5D-L–health**			.38
	Pre	90.42 (5.42)	
	Mid	86.33 (9.95)	
	Post	88.75 (6.78)	
**Test of Attentional Performance (total)**			.005
	Pre	5.04 (0.82)	
	Post	4.41 (0.63)	
**Test of Attentional Performance (positive)**			.001
	Pre	4.98 (0.76)	
	Post	4.41 (0.73)	
**Test of Attentional Performance (negative)**			.71
	Pre	5.44 (2.47)	
	Post	5.19 (1.53)	

a *P* value <.05 is significant.

b Trail Making Test–A is the selective attention test set.

c Trail Making Test–B is the divided attention test set.

Correlation analyses showed a significant positive association between participants’ fondness to read computer magazines and the search time slope (*r*=0.97; *P*<.001) and the slope of path time (*r*=0.75; *P*=.01). Both the search time slope (*r*=−0.83; *P*=.003) and the slope of path time (*r*=−0.78; *P*=.007) were found to be negatively correlated with the IMI subscale “pressure/tension.”

**Figure 4.**
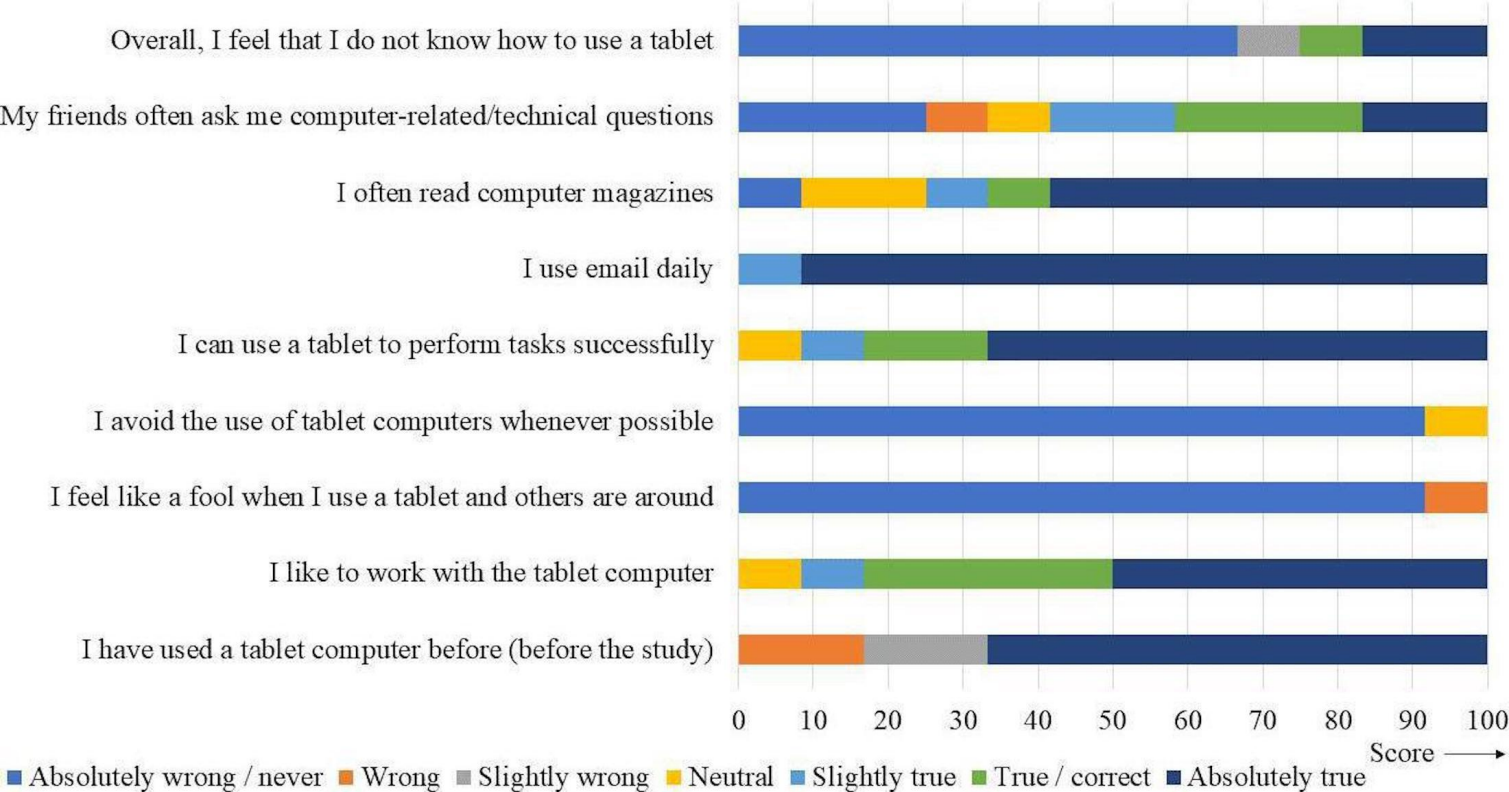
Overview of the tablet computer familiarity scores.

Of the 12 participants, 8 reported that they had used tablet computers before and could perform tasks successfully with them (Figure 4). Participants rated the IMI subscale “interest/enjoyment” with the highest score, while the “value/usefulness” was scored the lowest (Table 3). Participants scored physical demand the lowest compared to the other task load domains for the game intervention (Table 4).

**Table 3.** Intrinsic Motivation Inventory subscale scores reported by the participants.

Participant ID	Competence/effort	Interest/enjoyment	Value/usefulness	Pressure/tension
01	13	45	13	21
02	13	41	9	19
03	8	32	2	21
04	14	41	5	19
05	11	29	3	21
06	10	40	11	19
07	13	42	12	20
08	13	40	5	14
10	13	38	9	18
11	14	44	13	21
12	13	43	4	18
13	13	37	4	14

**Table 4.** Task Load Index subscale scores reported by the participants.

Participant ID	Mental demand	Physical demand	Temporal demand	Performance	Effort	Frustration
01	6	1	1	2	1	3
02	6	1	3	4	2	3
03	4	1	18	0	7	9
04	8	1	4	2	3	5
05	10	2	6	4	4	2
06	12	3	12	2	14	3
07	14	2	4	2	4	2
08	5	1	2	1	4	3
10	7	1	3	4	4	3
11	15	1	1	5	11	1
12	2	1	5	20	1	1
13	10	1	10	15	8	2

## Discussion

### Principal Findings

Puzzle games have the potential to target the following cognitive domains: learning and memory (working memory), attention (visual search), executive functions (inhibition and flexibility), and perceptual motor function (visuospatial ability). This study aimed to assess the feasibility of using a tablet-based puzzle game intervention to improve cognitive function and well-being.

We focused on associations between attention executive functions and game performance measures. The results of this pilot study show improvements in visual attention and visuospatial measures after the intervention.

### Limitations

The main limitation is the small sample size, which poses a risk of providing false significant results. If rigorously tested and evaluated in larger cohorts, it will help to increase the methodological rigor, amplify the transferability, and thereby enhance the specificity and sensitivity of the puzzle game as a diagnostic tool. The other limitation was the duration of our pilot study, which was not long enough. The successive study should be longer to measure a far transfer as well as minimize novelty effects in the data. The IMI scale was assessed at posttest (in the control group after the puzzle game intervention and in the experimental group after the newspaper-reading task) and might be a potential bias in the study. The carryover design and having no washout period owing to a carryover bias can be a possible shortcoming. Although reading a newspaper is a good control for cognitive tasks, it can be counterintuitive, thereby increasing anxiety about mental health outcomes. Therefore, it would be advisable to have an alternate control group engaged in other control activities such as playing games, which are readily accessible on various tablet applications and easily downloadable.

### Comparison With Prior Work

Visuospatial reasoning has been reported as a marker of Alzheimer disease and mild cognitive impairment [47]. Our results of improvement in the visual-scanning attentional and visuospatial measures also align with earlier studies where video games have been shown to benefit fluid cognitive abilities [48]. Fluid cognitive abilities are subject to age-related cognitive decline and crucial in activities of daily living, and hence need to be a focus in cognitive studies aiming for quality of life improvements [49].

However, most RCTs only investigate improvements in cognitive function. In our pilot study, we have attempted to evaluate the efficacy of training on cognition, emotional well-being, and quality of life.

A key aspect of motivational and enriching gameplay is the ability to continuously learn and adapt to new challenges. Dynamic adjustment systems can help to adapt to higher levels or to predict when the player is failing repeatedly to avoid such states. Random difficulty adjustment does not consider the player and can lead to unbalanced and unpredictable difficulty. Much of the existing dynamic level adjustment systems rely upon prediction. AI-based algorithms are used to follow these trends in predicting the time and level. The dynamic and personalized adjustment used in this study also uses a data-driven behavior that emerges from interaction with the game. The agent uses previous participant training data to learn and work out the prediction strategy. By using a person-centric adaption of the difficulty level, we were able to not only accommodate the heterogeneity in cognitive ability between participants but also continuously match the game difficulty to the skill level of the participant, thus benefitting the training effect and long-term motivation. Based on our results, it was also shown that participants with good visual search and executive skills progress faster to higher levels. Previous studies have demonstrated the effectiveness of video games for promoting multiple cognitive domains [5,50-53], quality of life [54], and emotional skills [55,56]. However, in our study, we didn’t find any significant difference in quality of life and well-being. The possible reason could be a small sample size, and future studies on a larger cohort might show significant changes in these variables.

A significant positive association between the participant’s fondness for reading computer magazines and puzzle game improvement indicates a high level of interest in technology and gaming. When individuals engage in reading computer magazines, they are exposed to similar cognitive challenges such as understanding technical concepts or following complex instructions. This mental exercise can transfer to puzzle games, improving their ability to think critically, solve puzzles more efficiently, and adapt to different game scenarios. When individuals are passionate about a subject, they tend to invest more time and effort into it. Consequently, the more engaged individuals are, the more opportunities they have to practice and improve their puzzle-solving skills, which can lead to better performance over time.

Research is lacking evidence of the long-term effects of such interventions, particularly if learning and improvement are meant to be lasting. The crossover design of this study minimizes the possibility of a carryover effect being a possible avenue of exploration. The sample size was heterogeneous, meaning the game applications were tested on a broad group of users. Previous studies have suggested cognitive decline begins at the age of 45 years [57,58]. Therefore, including a broader age range of adults (aged ≥45 years) in our study allowed for a better understanding of cognitive decline across different age groups and enhanced the research findings’ applicability to a wider range of individuals.

### Conclusions

Digital games are a feasible way to train cognition while allowing additional data for assessment and monitoring progress. Gameplay can induce neuroplastic reorganization that leads to long-term retention and transfer of skill. However, the two games used in the training intervention targeted different cognitive domains. Some participants can have excellent spatial navigation skills, while others are good at visual divided attention tasks. The benefit of games on players who are already good in the domains targeted by the game is questionable. Future studies should be longitudinal and target dedicated domains to avoid ceiling effects.

## Supplementary material

10.2196/46177 Checklist 1 CONSORT-eHEALTH checklist (V 1.6.1).

## Abbreviations

AI: artificial intelligence
CVG: casual video game
IMI: Intrinsic Motivation Inventory
MoCA: Montreal Cognitive Assessment
RCT: randomized controlled trial
REST: representational state transfer
TAP: Test of Attentional Performance
TMT: Trail Making Test
