# Supplementary material for: Tablet-Based Puzzle Game Intervention for Cognitive Function and Well-Being in Healthy Adults: Pilot Feasibility Randomized Controlled Trial
Source: JMIR Aging. 2023 Nov 1;6:e46177. doi: 10.2196/46177 (PMC10632698; doi:10.2196/46177)
Supplement: Checklist 1 [file aging-v6-e46177-s001.pdf]

| CONSORT-EHEALTH Checklist V1.6.2 Report | Manuscript Number | 46177 |
|---|---|---|
| **(based on CONSORT-EHEALTH V1.6), available at [http://tinyurl.com/consort-ehealth-v1-6].** | | |
| | | |
| **Date completed** | | |
| 9/9/2023 8:21:39 | | |
| **by** | | |
| Prabitha Urwyler | | |
| | | |
| Tablet-Based Puzzle Game Training Intervention on Cognitive Function and Well-Being in Healthy Adults Including Elderly People: Pilot Feasibility Randomized Controlled Trial | | |
| TITLE | | |
| **1a-i) Identify the mode of delivery in the title** | | |
| "Tablet-Based Puzzle Game" | | |
| **1a-ii) Non-web-based components or important co-interventions in title** | | |
| "Tablet-based" is self-explanatory" | | |
| **1a-iii) Primary condition or target group in the title** | | |
| "in Healthy Adults Including Elderly People" | | |
| ABSTRACT | | |
| **1b-i) Key features/functionalities/components of the intervention and comparator in the METHODS section of the ABSTRACT** | | |
| "was a randomized controlled trial using a crossover design with two phases (eight weeks each) and three measurement waves (pre, mid, and post). The participants were randomly allocated either to the control or experimental group. In the control group, participants read newspapers between the pre- and mid-test, then switched to cognitive training with puzzle games. In the experimental group, interventions were crossed over. Baseline measurements (pre-test) were collected prior to the intervention. " | | |
| **1b-ii) Level of human involvement in the METHODS section of the ABSTRACT** | | |
| "The interventions were delivered on tablet computers and took place unsupervised at participants' homes" | | |
| **1b-iii) Open vs. closed, web-based (self-assessment) vs. face-to-face assessments in the METHODS section of the ABSTRACT** | | |
| "The participants were randomly allocated either to the control or experimental group" | | |
| **1b-iv) RESULTS section in abstract must contain use data** | | |
| "The participants showed improvements in their visual attention and visuospatial measures after the puzzle game intervention" | | |
| **1b-v) CONCLUSIONS/DISCUSSION in abstract for negative trials** | | |
| "The study showed that digital games are a feasible way to train cognition in healthy adults and the elderly people" | | |
| INTRODUCTION | | |
| **2a-i) Problem and the type of system/solution** | | |
| "The use of video games to train strategic control in structured conditions may enhance cognition of both adult and elderly people " | | |
| **2a-ii) Scientific background, rationale: What is known about the (type of) system** | | |
| "Previous literature demonstrated artificial intelligence (AI)-based intervention as a promising tool for enhancing cognition, quality of life, and/or well-being among the elderly [21-23]. A recent large-scale study showed that regular engagement in Sudoku and similar puzzles represent a cognitively enriching leisure activity that prevents and delays age-related cognitive decline [24]. Several studies suggested puzzle video game sensitivity to the cognitive or motor alternations of normal ageing [25, 26]." | | |
| **Does your paper address CONSORT subitem 2b?** | | |

| | | |
|---|---|---|
| "The study aimed to conduct a pilot randomized clinical trial (P-RCT) to evaluate the potential benefits of the puzzle game intervention in healthy older adults including elderly. The primary objective was to examine whether a puzzle game supported by artificial intelligence (AI) to personalise the intervention, significantly improves attentional function (visual search attention) and leads to in-game learning effects. Other secondary objectives were to investigate improvements in further cognitive outcome measures proposed to be engaged by the puzzle game (attention, processing speed, working memory, and spatial reasoning) and the efficacy of the puzzle game intervention in reducing symptoms of depression, anxiety, and stress and improving everyday function and quality of life. Firstly, we expected significant improvement in attentional and executive function (near transfer) and other cognitive functions engaged by the game (far transfer). Secondly, we generalized that well-being (mood and stress) will show significant improvement" | | |
| METHODS | | |
| **3a) CONSORT: Description of trial design (such as parallel, factorial) including allocation ratio** | | |
| "The pilot was designed as a 16-weeks, randomized, controlled trial using a crossover design with two phases (8 weeks each) and three waves (pre, mid, and post) of measurement. The participants were randomly allocated either to the control or experimental group. Baseline measurements (pre-test) were collected prior to the intervention. In the control group, participants read newspapers at least 3 times a week for 8 weeks between the pre- and mid-test, then switched to cognitive training with puzzle games after the mid-test. In the experimental group, interventions were crossed over compared to the control group. The interventions were delivered on tablet computers (10.2" Apple iPad 2019 model, 32 GB, 4G edition (for sim card), Apple Inc., Cupertino, CA, USA,) and took place unsupervised at participants' homes (Figure 1). " | | |
| **3b) CONSORT: Important changes to methods after trial commencement (such as eligibility criteria), with reasons** | | |
| The measurements were maintained, however the number of subjects was not maintained. | | |
| **3b-i) Bug fixes, Downtimes, Content Changes** | | |
| | | |
| **4a) CONSORT: Eligibility criteria for participants** | | |
| "The inclusion criteria for participation were adults aged between 45 and 75 years and MoCA score≥24. The exclusion criteria for participation were any previous history of comorbid neurological or psychiatric deficits, any previous diagnosis of mild or major neurocognitive disorder, and insufficient coordinative, motor, and perceptual ability to handle a tablet computer. All participants had a normal or corrected-to-normal vision". | | |
| **4a-i) Computer / Internet literacy** | | |
| We used a computer familiarity questionnaire at the end of the study. | | |
| **4a-ii) Open vs. closed, web-based vs. face-to-face assessments:** | | |
| "The sample included healthy older adults and the elderly people (n=12; 6 female; mean age=58.92 [SD 10.28] years; range: 46 to 75 years), recruited from the local community." | | |
| **4a-iii) Information giving during recruitment** | | |
| "A written informed consent form was sought from each participant before participation."<br>"After completing recruitment formalities, participants were given an introduction to the study followed by a training session of both the games consisting of 4 trails each of three grid sizes (4x4, 5x5, 6x6)at least one level for each grid size." | | |
| **4b) CONSORT: Settings and locations where the data were collected** | | |
| "At three-time points (pre-, mid-, and post-test) global cognitive function, higher cognitive function, and emotional well-being were assessed using standardized neuropsychological tests (computerized visual scanning Test of attentional performance TAP task [30], Montreal Cognitive Assessment (MoCA) [31], Trail Making Test (TMT) [32], Snellgrove Maze Test (SMT) [33], and questionnaires (Profile of Mood States- POMS) [34-36] , State-Trait Anxiety Inventory- STAI) [37, 38], and quality of life questionnaire [39]). To remove bias in too-close measurements, alternative versions of MoCA were used in pre, mid, and post-assessments. Additionally, information on the cognitive load (NASA Task Load Index [40]), motivation (adapted version of Intrinsic Motivation Inventory [41]), and familiarity with tablet usage (adapted Tablet Familiarity Questionnaire [42]) were collected at the end of the study. " | | |
| **4b-i) Report if outcomes were (self-)assessed through online questionnaires** | | |
| "All assessments were administered in paper-pencil format except for the computerized visual scanning TAP task presented on a laptop" | | |
| **4b-ii) Report how institutional affiliations are displayed** | | |
| The affiliations were only seen in the Ethic consent form i.e. after recruitment | | |
| **5) CONSORT: Describe the interventions for each group with sufficient details to allow replication, including how and when they were actually administered** | | |
| **5-i) Mention names, credential, affiliations of the developers, sponsors, and owners** | | |
| "The study was partially supported by a Novartis–Freenovation grant to P. Urwyler. The funders had no role in study design, data collection and analysis, decision to publish, or preparation of the manuscript." "The authors declare no conflicts of interest." | | |

| | | |
|---|---|---|
| **5-ii) Describe the history/development process** | | |
| "Particularly maze-like Numberlink (NL) and match-3 (M3) puzzle video game performance was shown as a strong predictor of assessing cognitive or motor variabilities in older adults [25, 27]. These games can be varied in difficulty to match the users' level of cognitive ability can help prevent practice effects during repeated administration and reduce ceiling and flooring effects by continuously matching the task difficulty to the participant's cognitive ability level [28, 29]. " | | |
| **5-iii) Revisions and updating** | | |
| As the version was frozen, we did not report it. | | |
| **5-iv) Quality assurance methods** | | |
| | | |
| **5-v) Ensure replicability by publishing the source code, and/or providing screenshots/screen-capture video, and/or providing flowcharts of the algorithms used** | | |
| | | |
| **5-vi) Digital preservation** | | |
| | | |
| **5-vii) Access** | | |
| The participants obtained a reimbursement for participating in the study. "Participants were instructed to play a minimum of 3 times a week (max. 10 min/game) both puzzle games (M3, NL) delivered to them on an iPad. " | | |
| **5-viii) Mode of delivery, features/functionalities/components of the intervention and comparator, and the theoretical framework** | | |
| "Participants were instructed to play a minimum of 3 times a week (max. 10 min/game) both puzzle games (M3, NL) delivered to them on an iPad. A time-tracker in the game limited the participants' daily training time to 20 minutes, while a score-boarder displayed stars corresponding to the number of levels completed by the participants" | | |
| **5-ix) Describe use parameters** | | |
| "Participants were instructed to play a minimum of 3 times a week (max. 10 min/game) both puzzle games (M3, NL) delivered to them on an iPad. A time-tracker in the game limited the participants' daily training time to 20 minutes, while a score-boarder displayed stars corresponding to the number of levels completed by the participants" | | |
| **5-x) Clarify the level of human involvement** | | |
| | | |
| **5-xi) Report any prompts/reminders used** | | |
| "The AI server logged every request sent from the iPad to the server."<br><br>This is unsupervised training, and the subject is allowed to train whichever time he/she wishes. A mail alerting service using the free tier of mailgun was implemented to follow up with the daily progress on the subject's gameplay. A log file on the server was generated with of the number, type of requests per user. This log file was sent to the project admin and research assistants to keep a vigil on the study participants. The participants were contacted per telephone when the non-activity period reached seven days. | | |
| **5-xii) Describe any co-interventions (incl. training/support)** | | |
| There were no co-interventions in addition to the main intervention | | |
| **6a) CONSORT: Completely defined pre-specified primary and secondary outcome measures, including how and when they were assessed** | | |
| "At three-time points (pre-, mid-, and post-test) global cognitive function, higher cognitive function, and emotional well-being were assessed using standardized neuropsychological tests (computerized visual scanning Test of attentional performance TAP task [30], Montreal Cognitive Assessment (MoCA) [31], Trail Making Test (TMT) [32], Snellgrove Maze Test (SMT) [33], and questionnaires (Profile of Mood States- POMS) [34-36], State-Trait Anxiety Inventory- STAI) [37, 38], and quality of life questionnaire [39])." | | |
| **6a-i) Online questionnaires: describe if they were validated for online use and apply CHERRIES items to describe how the questionnaires were designed/deployed** | | |
| | | |
| **6a-ii) Describe whether and how "use" (including intensity of use/dosage) was defined/measured/monitored** | | |
| | | |

| | | |
|---|---|---|
| **6a-iii) Describe whether, how, and when qualitative feedback from participants was obtained** | | |
| Feedback was obtained through email and telephone | | |
| **6b) CONSORT: Any changes to trial outcomes after the trial commenced, with reasons** | | |
| "At three-time points (pre-, mid-, and post-test) global cognitive function, higher cognitive function, and emotional well-being were assessed using standardized neuropsychological tests (computerized visual scanning Test of attentional performance TAP task [30], Montreal Cognitive Assessment (MoCA) [31], Trail Making Test (TMT) [32], Snellgrove Maze Test (SMT) [33], and questionnaires (Profile of Mood States- POMS) [34-36] , State-Trait Anxiety Inventory- STAI) [37, 38], and quality of life questionnaire [39]). To remove bias in too-close measurements, alternative versions of MoCA were used in pre, mid, and post-assessments. Additionally, information on the cognitive load (NASA Task Load Index [40]), motivation (adapted version of Intrinsic Motivation Inventory [41]), and familiarity with tablet usage (adapted Tablet Familiarity Questionnaire [42]) were collected at the end of the study. " | | |
| **7a) CONSORT: How sample size was determined** | | |
| **7a-i) Describe whether and how expected attrition was taken into account when calculating the sample size** | | |
| Attrition was taken into account when calculating the sample size | | |
| **7b) CONSORT: When applicable, explanation of any interim analyses and stopping guidelines** | | |
| “At three-time points (pre-, mid-, and post-test) global cognitive function, higher cognitive function, and emotional well-being were assessed using standardized neuropsychological tests (computerized visual scanning Test of attentional performance TAP task [30], Montreal Cognitive Assessment (MoCA) [31], Trail Making Test (TMT) [32], Snellgrove Maze Test (SMT) [33], and questionnaires (Profile of Mood States- POMS) [34-36], State-Trait Anxiety Inventory- STAI) [37, 38], and quality of life questionnaire [39]).” | | |
| **8a) CONSORT: Method used to generate the random allocation sequence** | | |
| “The pilot was designed as a 16-weeks, randomized, controlled trial using a crossover design with two phases (8 weeks each) and three waves (pre, mid, and post) of measurement. The participants were randomly allocated either to the control or experimental group.” | | |
| **8b) CONSORT: Type of randomisation; details of any restriction (such as blocking and block size)** | | |
| It does not apply to our study due to a small sample size. | | |
| **9) CONSORT: Mechanism used to implement the random allocation sequence (such as sequentially numbered containers), describing any steps taken to conceal the sequence until interventions were assigned** | | |
| The random allocation sequence was generated by drawing lots of two groups (control and experiment) and participants were asked to pick any one lot randomly. | | |
| **10) CONSORT: Who generated the random allocation sequence, who enrolled participants, and who assigned participants to interventions** | | |
| The allocation sequence, enrolment of the participants and assigned participants to interventions were performed by the master students under the supervision of the Principal Investigator Dr. Prabitha Urwyler | | |
| **11a) CONSORT: Blinding - If done, who was blinded after assignment to interventions (for example, participants, care providers, those assessing outcomes) and how** | | |
| **11a-i) Specify who was blinded, and who wasn’t** | | |
| In our study, both researchers and participants were blinded about the allocation of the groups | | |
| **11a-ii) Discuss e.g., whether participants knew which intervention was the “intervention of interest” and which one was the “comparator”** | | |
| | | |
| **11b) CONSORT: If relevant, description of the similarity of interventions** | | |
| Since our study has only a single intervention (puzzle game), therefore, it does not apply to our study. | | |
| **12a) CONSORT: Statistical methods used to compare groups for primary and secondary outcomes** | | |
| "The normality of all values of interest was checked using the Shapiro-Wilk test [46]. An alpha value of 0.05 was used to determine significance. To evaluate the intervention effect on subjects’ cognitive function and emotional well-being at three-time points (pre, mid, and post), repeated-measure ANOVA was performed. Paired t-tests were used to compare the visual search attention (visual scanning TAP) before and after the intervention wave. Correlation analyses were used to examine the intervention performance and efficiency and the cognitive and emotional measures." | | |
| **12a-i) Imputation techniques to deal with attrition / missing values** | | |
| "The data of a participant (subject ID 9) was excluded due to the incomplete measurement of subjects’ cognitive function and emotional well-being at three-time points (pre, mid, and post)." | | |
| **12b) CONSORT: Methods for additional analyses, such as subgroup analyses and adjusted analyses** | | |
| Due to the small sample size, it does not apply to our study | | |
| RESULTS | | |

| | | |
|---|---|---|
| **13a) CONSORT: For each group, the numbers of participants who were randomly assigned, received intended treatment, and were analysed for the primary outcome** | | |
| “The participants were randomly allocated either to the control or experimental group. Baseline measurements (pre-test) were collected prior to the intervention. In the control group, participants read newspapers at least 3 times a week for 8 weeks between the pre- and mid-test, then switched to cognitive training with puzzle games after the mid-test. In the experimental group, interventions were crossed over compared to the control group.” | | |
| **13b) CONSORT: For each group, losses and exclusions after randomisation, together with reasons** | | |
| The data of a participant (subject ID 9) was excluded due to the incomplete measurement of subjects’ cognitive function and emotional well-being at three-time points (pre, mid, and post). | | |
| **13b-i) Attrition diagram** | | |
| | | |
| **14a) CONSORT: Dates defining the periods of recruitment and follow-up** | | |
| The recruitment and assessment at different time points were performed between March 2020 to Feb 2021 | | |
| **14a-i) Indicate if critical “secular events” fell into the study period** | | |
| | | |
| **14b) CONSORT: Why the trial ended or was stopped (early)** | | |
| Since it was a pilot and time-bound study. Therefore, with the possible number of participants within the time limit we completed our study. | | |
| **15) CONSORT: A table showing baseline demographic and clinical characteristics for each group** | | |
| "The demographics and characteristics of the subjects included in this pilot study are shown in table 1. " | | |
| **15-i) Report demographics associated with digital divide issues** | | |
| All the participants were recruited from the local community with similar demographic characteristics, therefore, digital divide issues were not applicable to our study. | | |
| **16a) CONSORT: For each group, number of participants (denominator) included in each analysis and whether the analysis was by original assigned groups** | | |
| **16-i) Report multiple “denominators” and provide definitions** | | |
| In our study, all participants consented and completed the study as per the study protocols. Therefore, there is no denominator is applicable to our study. | | |
| **16-ii) Primary analysis should be intent-to-treat** | | |
| | | |
| **17a) CONSORT: For each primary and secondary outcome, results for each group, and the estimated effect size and its precision (such as 95% confidence interval)** | | |
| We did not perform effect size calculation, due to the small sample size. | | |
| **17a-i) Presentation of process outcomes such as metrics of use and intensity of use** | | |
| | | |
| **17b) CONSORT: For binary outcomes, presentation of both absolute and relative effect sizes is recommended** | | |
| It is not applicable because our study outcome is not binary outcome | | |
| **18) CONSORT: Results of any other analyses performed, including subgroup analyses and adjusted analyses, distinguishing pre-specified from exploratory** | | |
| Due to the small sample size, it does not apply to our study | | |
| **18-i) Subgroup analysis of comparing only users** | | |
| | | |
| **19) CONSORT: All important harms or unintended effects in each group** | | |
| Although reading a newspaper is a good control for cognitive tasks, they can be counter-intuitive thereby increasing anxiety for mental health outcomes.” | | |
| **19-i) Include privacy breaches, technical problems** | | |
| | | |
| **19-ii) Include qualitative feedback from participants or observations from staff/researchers** | | |
| We had a general verbal feedback from the participants | | |
| DISCUSSION | | |
| **20) CONSORT: Trial limitations, addressing sources of potential bias, imprecision, multiplicity of analyses** | | |

| | | |
|---|---|---|
| **20-i) Typical limitations in ehealth trials** | | |
| "The main limitation is the small sample size, which poses a risk of false significant results. If rigorously tested and evaluated in larger cohorts, it will help to increase methodological rigor, amplify the transferability, and thereby enhance the specificity and sensitivity of the puzzle game as a diagnostic tool. The other limitation was that the duration of our pilot study was not long enough. The successive study should be long enough to measure a far transfer as well as minimise novelty effects in the data. The IMI scale was assessed at post-test (in the control group after the puzzle game intervention, while in the experimental group after the newspaper reading task) and might be a potential bias in the study. The carry-over design plus no wash out period owing to a carry-over bias can be a possible shortcoming. Although reading a newspaper is a good control for cognitive tasks, they can be counter-intuitive thereby increasing anxiety for mental health outcomes. " "The possible reason could be a small sample size and future studies on a larger cohort might show significant changes in these variables." | | |
| **21) CONSORT: Generalisability (external validity, applicability) of the trial findings** | | |
| **21-i) Generalizability to other populations** | | |
| "The results of the study will guide future prevention efforts and trials in high-risk populations." | | |
| **21-ii) Discuss if there were elements in the RCT that would be different in a routine application setting** | | |
| | | |
| **22) CONSORT: Interpretation consistent with results, balancing benefits and harms, and considering other relevant evidence** | | |
| **22-i) Restate study questions and summarize the answers suggested by the data, starting with primary outcomes and process outcomes (use)** | | |
| "We focused on associations between attention executive functions and game performance measures. The results of this pilot study show improvements in visual attention and visuospatial measures after the intervention. " | | |
| **22-ii) Highlight unanswered new questions, suggest future research** | | |
| "It would be advisable to have an alternate control group engaged in other control activities such as playing games, which are readily accessible on various tablet applications and easily downloadable." "Future studies should be longitudinal in nature and target dedicated domains to avoid ceiling effects" | | |
| Other information | | |
| **23) CONSORT: Registration number and name of trial registry** | | |
| This study is approved by the Ethics Committee of Bern (ID 2016-01281) and Northwest/Central Switzerland.<br>Trial registration: ClinicalTrials.gov (NCT03139799) | | |
| **24) CONSORT: Where the full trial protocol can be accessed, if available** | | |
| "https://clinicaltrials.gov/study/NCT03139799" | | |
| **25) CONSORT: Sources of funding and other support (such as supply of drugs), role of funders** | | |
| "The funders had no role in study design, data collection and analysis, decision to publish, or preparation of the manuscript" | | |
| **X26-i) Comment on ethics committee approval** | | |
| "Ethical approval was granted by the University of Bern ethics committee (ID 2016-01281) and the Ethics Committee Northwest/ Central Switzerland. The study was carried out following the current version of the Declaration of Helsinki, the guidelines of GCP" | | |
| **x26-ii) Outline informed consent procedures** | | |
| "A written informed consent form was sought from each participant before participation." | | |
| **X26-iii) Safety and security procedures** | | |
| | | |
| **X27-i) State the relation of the study team towards the system being evaluated** | | |
| The authors are distinct from the sponsors of the intervention. | | |
